# The value of DHR-enzyme-linked immunosorbent assay in the diagnosis of chronic granulomatous disease by detecting NADPH oxidase complex activity

**DOI:** 10.3389/fped.2025.1483173

**Published:** 2025-06-05

**Authors:** Wen Xiaohui, Zhao Shunying, Zhang Xiaoyan, Tang Xiaolei, Shen Yuelin, Liu Jinrong, Li Huimin, Liu Hui, Yang Haiming

**Affiliations:** Department No. 2 of Respiratory Medicine, National Children’s Medical Center, Beijing Children’s Hospital, Capital Medical University, Beijing, China

**Keywords:** chronic granulomatous disease, children, DHR-enzyme-linked immunosorbent assay, NADPH oxidase complex activity detection, respiratory burst

## Abstract

**Background:**

A rapid, easy, and accurate method for screening Chronic Granulomatous Disease (CGD) is crucial. This study aimed to propose and evaluate the effectiveness of the DHR-Enzyme-linked immunosorbent assay (DHR-ELISA) for assessing NADPH oxidase complex activity in the early screening of CGD.

**Methods:**

We conducted a retrospective analysis of 72 children suspected of having CGD who underwent NADPH oxidase activity assessment and genetic testing at Beijing Children's Hospital between July 2015 and January 2022.

**Results:**

Of the subjects, 57 were male and 15 were female, resulting in a male-to-female ratio of 3.8:1. The median age at onset was 6 months, and the median age at diagnosis was 15 months. Thirty-eight patients were diagnosed with CGD based on typical clinical manifestations and genetic testing, exhibiting symptoms such as left lymphadenopathy or calcification (65.8%), a large Calmette-Guérin scar (60.5%), a history of skin or other lymph node infections (52.6%), and specific pulmonary infections (23.7%). Thirty-one patients exhibited normal enzyme activity, whereas 41 showed reduced activity. The DHR-ELISA method demonstrated a specificity of 90% and a sensitivity ranging from 90.5% to 100% in detecting CGD.

**Conclusion:**

The DHR-ELISA is a rapid, easy, cost-effective, and efficient method for screening CGD, making it suitable for early diagnosis and potentially improving prognosis.

## 1 Introduction

Chronic granulomatous disease (CGD) was initially described by Janeway in 1954 and later characterized as “fatal childhood granulomatous disease” in 1959 (1). It constitutes a rare inborn error of immunity. The estimated prevalence of CGD is approximately 1 in 200,000–250,000 live births in the United States (2). Incidence rates vary among different ethnic groups, and precise data for China have yet to be established. Advances in treatment have significantly improved the overall survival rates for patients with CGD. However, in China, the disease is characterized by early onset and delayed diagnosis. Our team has identified eight diagnostic indicators that facilitate the early detection of CGD in Chinese patients, aiding clinicians in timely disease recognition (3). Patients suspected of having CGD require comprehensive diagnostic testing to confirm the diagnosis. Given that genetic testing is time-consuming, costly, and occasionally inconclusive, the dihydrorhodamine 123 (DHR) flow cytometric assay is recognized as the standard screening method due to its rapidity and high efficiency. However, the DHR-flow cytometric assay is unsuitable for primary hospitals lacking flow cytometry equipment. We have found that the DHR-Enzyme-linked immunosorbent assay (DHR-ELISA) can be used to evaluate the activity of the nicotinamide adenine dinucleotide phosphate (NADPH) oxidase complex in neutrophils, offering significant benefits in terms of diagnostic efficiency, cost, and detection time, thereby meriting broader application.

## 2 Materials and methods

### 2.1 Participant profile

A cohort of 72 pediatric patients suspected of having CGD was subjected to an assessment for CGD using the DHR-ELISA method to evaluate NADPH oxidase complex function and whole exome sequencing (WES) with parental segregation analysis (07/2015–01/2022). This study was approved by the Ethics Committee of Beijing Children's Hospital (No.2024-E-012-R).

### 2.2 Indicators for CGD suspicion

Based on literature, eight indicators have been established for suspecting CGD, which include a large BCG scar, left axillary lymphadenopathy or calcification, skin or other lymph node infections, skin scars, multiple lung nodules, perianal abscesses, pulmonary *Aspergillus* infection and *Burkholderia* infection, and repetitive respiratory infections (3, 4).

### 2.3 CGD diagnostic benchmarks

Patients presenting with symptoms indicative of chronic granulomatous disease and possessing any variants in the *CYBB*, *CYBA*, *NCF1*, *NCF2*, *NCF4*, or *CYBC1* gene, as confirmed by WES, with the variant's pathogenicity classified as either pathogenic or likely pathogenic according to the ACMG clinical practice guideline, meet the diagnostic criteria for CGD.

### 2.4 DHR-ELISA of NADPH oxidase complex activity

Fresh EDTA-anticoagulated leukocytes from patients were collected and diluted 10 times with a buffer solution (PBS). Then, 80 μl of this diluted solution was added to a fluorescence detection enzyme plate. Next, 2 μl of dihydrorhodamine 123 (DHR123, 0.5 mg/ml, Thermo) was mixed in and incubated at 37°C for 5 min. Subsequently, 1 μl of PMA (100 μg/ml, Sigma) was added and incubated again at 37°C for 30 min. The fluorescence intensity was measured with an ELISA reader. The results were expressed in terms of enzymatic activity (F/μg) and relative enzymatic activity (the ratio of the sample's enzymatic activity to the average normal enzymatic activity). The reference range (1,332–9,312 F/μg) was established by testing 100 healthy individuals, yielding a mean NADPH oxidase activity of 4,311 F/μg (±2 SD). Relative enzymatic activity spanned 31%–216%. Quality control performed in accordance with the ELISA methodology. The reliability of our assay was confirmed by testing 5 patients with CGD and their parents using DHR-ELISA and DHR-flow cytometry, with both methods showing concordant results.

### 2.5 Statistical analysis

Clinical profiles and laboratory test outcomes were gathered from an extensive review of hospitalized cases, outpatient records, and telephonic follow-ups for the subjects. Data were presented as median values with interquartile ranges [*M* (P25, P75)]. Analyses of specificity and sensitivity regarding the NADPH oxidase complex activity, as determined by DHR-ELISA, and the confirmation of CGD were conducted.

The study was approved by the Ethics Committee of Beijing Children's Hospital, Beijing, China. Written consent was obtained from the parents of the study subjects.

## 3 Results

### 3.1 Demographic profile

Among the group of 72 patients, there were 57 males and 15 females, resulting in a male-to-female ratio of approximately 3.8:1. The median age at initial presentation was 6 months, with an interquartile range (IQR) from 11 to 20 months. The average age at the time of diagnosis was 15 months, with an IQR spanning from 6 to 47 months. The admission figures were distributed as follows: 21 cases in 2015–2016, 29 cases in 2017–2018, 13 cases in 2019–2020, and 9 cases in 2021–2022.

### 3.2 Presentation in CGD-affected children

Of the 72 cases, 38 were confirmed to have CGD according to the CGD Diagnostic Benchmarks stated earlier. Among the 38 patients with CGD, 23 presented with extensive scarring (23/38, 60.5%), 25 had lymphadenopathy or calcification in the left axillary region (25/38, 65.8%), 9 had pulmonary infections attributed to specific pathogens (9/38, 23.7%), 3 had allergic alveolitis (3/38, 7.9%), 20 had skin or other lymph node infections (20/38, 52.6%), 4 had infections at miscellaneous sites (4/38, 10.5%), and 2 had hemophagocytic lymphohistiocytosis (2/38, 5.3%), as detailed in Table 1.

**Table 1:** Distribution of clinical manifestations in 38 cases of CGD.

Clinical manifestations	Distribution of cases and percentages
Large BCG scar	23 (23/38, 60.5%)
Left axillary lymphadenopathy or calcification	25 (25/38, 65.8%)
Pulmonary *aspergillosis* infection	5 (5/38, 13.2%)
Pulmonary *Burkholderia* infection	3 (3/38, 7.9%)
Pulmonary *Nocardia* infection	1 (1/38, 2.6%)
Allergic alveolitis	3 (3/38, 7.9%)
Skin scar	6 (6/38, 15.8%)
Perianal abscess	10 (10/38, 26.3%)
Lymphadenitis at other sites	8 (8/38, 21.1%)
Sepsis	3 (3/38, 7.9%)
Osteomyelitis	1 (1/38, 2.6%)
Hemophagocytic lymphohistiocytosis	2 (2/38, 5.3%)

BCG, Bacillus Calmette-Guérin vaccine.

### 3.3 WES findings in CGD

Thirty-eight patients were diagnosed with CGD according to the CGD Diagnostic Benchmarks mentioned above. Among this cohort of 38 patients with CGD, mutations in the *CYBB* gene were identified in 27 individuals (27/38, 71.1%). Mutations in the *NCF1* gene were present in 6 children (6/38, 15.8%), *CYBA* gene alterations were found in 3 patients (3/38, 7.9%), and *NCF2* gene mutations were detected in 2 cases (2/38, 5.3%), as presented in Table 2. Additionally, 4 patients exhibited atypical clinical signs of CGD, and their genetic testing revealed variants in genes associated with CGD; however, the pathogenicity level did not meet the criteria for a definitive diagnosis, thereby categorizing them as cases with uncertain significance, as detailed in Table 3.

**Table 2:** The distribution of mutant genes and the detection results of NADPH oxidase complex activity by DHR-ELISA in 38 children with CGD.

Mutant genes	Number of examples	Enzyme activity (F/ug)	Relative enzyme activity value (%)
*CYBB*	27 (27/38, 71.0%)	161 (110, 286)	4 (3, 7)
*NCF1*	6 (6/38, 15.8%)	157.5 (124, 242)	3.5 (3, 6)
*CYBA*	3 (3/38, 7.9%)	175 (158.5, 322.5)	4 (3.5, 7.5)
*NCF2*	2 (2/38, 5.3%)	339.5 (129, 550)	8 (3, 13)

**Table 3:** Information of 4 uncertain suspected CGD cases.

No.	Age at onset (months)	Sex	CGD clinical representations	Other diagnosis/phenotypes	Genetic test		DHR-ELISA	
					Causative genes	ACMG classification	Enzyme activity (F/μg)	Relative Enzyme Activity (%)
1	118	Male	Mediastinal lymph node tuberculosis	Tuberculous meningitis, serratia marcescens infection	NCF2 (compound heterozygous mutations)	Uncertain significance	5,064	117
2	48	Male	Recurrent respiratory infections	Elevated total serum IgE	CYBB	Uncertain significance	9,192	213
3	121	Male	Recurrent respiratory infections	Castleman's disease; bronchiolitis obliterans	CYBB	Benign	2,935	68
4	0.3	Male	Recurrent respiratory infections	Periodic fever	CYBB	Uncertain significance	1,528	35

### 3.4 Results of NADPH oxidase complex activity test

Among the 31 normal cases (including 4 cases detected with variants in CGD genes), the median value of enzyme activity units was 2,875 F/μg, with an interquartile range (IQR) of 2,286–3,705 F/μg; the median relative enzyme activity was 67%, with an IQR of 53%–86%. There were 41 cases with reduced results (3 of which were non-CGD patients), with a median enzyme activity unit of 170 F/μg and an IQR of 122.5–325 F/μg; the median relative enzyme activity was 4%, with an IQR of 3%–7.5%.

### 3.5 Analytical outcomes

Four individuals exhibiting normal DHR-ELISA assay readings, yet with genetic testing indicating the presence of CGD-associated genes, were categorized within the CGD-genetic sequencing group (refer to Table 4). The specificity (true negative fraction) was calculated at 90% (27/30), and the sensitivity (true positive fraction) at 90.5% (38/42). Excluding the four subjects with indeterminate CGD status, the specificity and sensitivity of the DHR-ELISA in assessing NADPH oxidase complex activity were maintained at 90% (27/30) and elevated to 100% (38/38), respectively.

**Table 4:** Distribution of the results of DHR-ELISA and genetic sequencing of CGD.

DHR-ELISA results	WES (+)	WES (−)	Total
Positive (reduced result)	38	3	41
Negative (normal result)	4	27	31
Total	42	30	72

WES (+), positive genetic sequencing of CGD; WES (−), negative genetic sequencing of CGD.

## 4 Discussion

Chronic granulomatous disease (CGD) is a rare human inborn error of immunity stemming from an anomaly in the subunits of the NADPH oxidase complex. This complex is composed of five protein subunits: two transmembrane proteins (gp91phox and p22phox, encoded by *CYBB* and *CYBA* genes, respectively) and three cytosolic proteins (p47phox, p67phox, and p40phox, encoded by the *NCF1*, *NCF2*, and *NCF4* genes, respectively). The primary mutations occur in the first four subunits. Mutations in the *CYBB* gene are inherited in an X-linked recessive manner, representing approximately 70% of cases, while the remaining mutations follow an autosomal recessive inheritance pattern. Moreover, the *CYBC1* gene encodes the Eros protein, essential for the formation of gp91phox-p22phox heterodimers, and mutations in *CYBC1* are also linked to CGD, presenting with diverse phenotypes (5, 6). Phagocytic cells (monocytes, macrophages, neutrophils, etc.) rely on the NADPH oxidase complex to generate superoxide anions and other reactive oxygen species during pathogen phagocytosis, a process termed the “respiratory burst.” The byproducts of this process, together with lysosomes, play a role in neutralizing the ingested microorganisms. Consequently, patients with CGD exhibit impairments in their phagocytic respiratory bursts, leading to recurrent, life-threatening bacterial and fungal infections, granuloma formation due to excessive inflammatory responses, and sometimes, autoimmune diseases. Compared to autosomal recessive CGD (AR-CGD), X-linked CGD manifests earlier and has a poorer prognosis (2); survival rates are closely associated with the production of reactive oxygen intermediates (R) (7).

Advances in treatment have notably increased the overall survival rates for patients with CGD (8). Statistical data indicate that nearly 90% of patients with CGD can reach early adulthood, with some undergoing bone marrow transplantation and others maintained on lifelong prophylactic medication (antibacterial, antifungal, and immunomodulatory agents) (9). However, the clinical presentation of patients with CGD is widely variable, affecting multiple organ systems such as the lungs, skin, lymph nodes, liver, bones, and gastrointestinal tract. Patients may be infected with various catalase-positive pathogens (e.g., *Staphylococcus aureus*, *Aspergillus*, *Serratia*, *Burkholderia*, *Nocardia*) and, in severe cases, may develop macrophage activation syndrome or hemophagocytic lymphohistiocytosis (4, 10–12), complicating the diagnosis. Our team suggests that events related to BCG vaccination, historical skin or site-specific lymph node infections, bilateral lung nodules, perianal abscesses, and infections by *Aspergillus* or *Burkholderia* (singly or in combination) serve as early diagnostic indicators for CGD in Chinese children (3), aiding clinicians in prompt identification and diagnosis through rapid, accurate, and cost-effective tests following a suspected CGD diagnosis.

The DHR-flow cytometric assay is the laboratory gold standard for screening CGD, with genetic testing available to confirm CGD when mutations are found in *CYBB*, *CYBA*, *NCF1*, *NCF2*, *NCF4*, and *CYBC1* (13). Although genetic testing can pinpoint mutations and aid in prognostication, it is not ideal for screening due to its cost, duration, and potential for indeterminate results. As a globally recognized standard, DHR-flow cytometric assays require samples to be preserved in EDTA tubes and analyzed preferably within 24–48 h (14, 15). They offer high sensitivity and a short testing cycle, operating on the principle that DHR123, after entering cells and oxidizing to rhodamine 123 (R123) in the mitochondria, emits green fluorescence upon blue light excitation (16). However, this method demands expensive equipment and skilled laboratory technicians, which many hospitals, particularly smaller ones, may lack, leading to delayed diagnosis. While the nitroblue tetrazolium (NBT) test, a cost-effective screening method for CGD, has been largely replaced by the DHR assay, it still requires the expertise of skilled laboratory technicians and is associated with numerous additional challenges (17). The DHR-ELISA method utilized in this study shares the same detection principle as DHR-flow cytometry but has lower operational requirements, demonstrating a specificity of 90% and a sensitivity between 90.5% and 100%. The low rates of misdiagnosis and missed diagnosis make it comparable to DHR-flow cytometry and thus suitable for widespread use. Additionally, the DHR-flow cytometric assay could be normal or mildly impaired in patients with NCF4/p40phox deficiency (18), and a similar result might occur with DHR-ELISA.

Three children exhibited clinical signs of left axillary lymphadenopathy and reduced DHR-ELISA results, yet genetic tests did not confirm CGD (one was diagnosed with Shwachman-Diamond syndrome, one showed no significant genetic abnormalities, and one was confirmed to have X-linked hyper IgM syndrome), indicating that DHR-ELISA assays may yield false positives. Previous reports suggest that false positives in DHR-flow cytometry may occur in cases lacking myeloperoxidase (19) or may be influenced by acetaminophen intake within 24 h prior to sampling (20), and acute disease phases can also lead to false positives (21). Therefore, caution is necessary when diagnosing suspected CGD in children with atypical clinical features and positive DHR-ELISA assays. In this study, four cases had normal DHR-ELISA results and lacked typical clinical signs of CGD at follow-up, with genetic tests revealing CGD-related genes but inadequate pathogenicity classification, thus classifying them as indeterminate. This suggests that residual NADPH enzyme function may account for the absence of clinical phenotype manifestation, necessitating continued monitoring and protein function testing for clarification, as current literature highlights that adult-diagnosed patients with CGD often exhibit milder phenotypes (22). Previous reports also indicate that low normal values in DHR-flow cytometry do not entirely exclude the possibility of CGD (17).

In summary, the DHR-ELISA technique, employed to assess NADPH oxidase complex function, stands out for its rapid processing time, affordability, simplicity in execution, and high efficacy in detection. This method is recommended as an initial screening approach for potential CGD cases and is particularly advantageous for broader implementation, including in primary healthcare settings lacking flow cytometric capabilities, to facilitate early CGD diagnosis and improve prognosis.

## Data Availability

The original contributions presented in the study are included in the article, further inquiries can be directed to the corresponding authors.
